# Phrenology—Its Nature and Uses; an Address to the Students of Anderson's University, at the Opening of Dr. Weir's First Course of Lectures on Phrenology in That Institution, Jan. 7, 1846

**Published:** 1846-07

**Authors:** 


Art. VII.—*Phrenology—Its Nature and Uses: an Address to the Students of Anderson's University, at the opening of Dr. Weir's first Course of Lectures on Phrenology in that Institution, Jan.* 7, 1846. By Andrew Combe, m.d.—*Edinburgh,* 1846. 8vo, pp. 32.

It will interest some of our readers to learn, from the title-page of Dr. Combe's Address, that a Chair of Phrenology has been instituted at Glasgow, in that flourishing school of medicine known as "Anderson's University." Respecting the propriety or the judiciousness of such a proceeding persons will differ, there is no doubt, according to the views which they entertain of phrenology itself. As on several former occasions, we have supported its claims to scientific investigation, believing it to embody much that is true, whatever may be the imperfections characterizing many of the details, we frankly avow ourselves to be gratified at the liberal step which the managers of the school in question have taken.

If we had not more substantial reasons for recommending this subject to the notice of the profession, one might yet be found in the fact that phrenology offers to the physiologist, at the very lowest on *plausible* grounds, a rational exposition of the functions of particular parts of the brain. This is a want in physiology that has long been felt; and if Gall have not, in some degree, supplied this desideratum, the labours of other physiologists have certainly not been more successful; for although theories have been propounded, none of them have exhibited any solid foundation in fact, nor yet, by their antecedent probability, have they been enabled to attach to themselves any considerable number of adherents. Phrenology, however, has for many years, and in many countries, been received and advocated by numerous able and estimable physiologists, as furnishing a true physiology of the brain; and we hold that, under such circumstances, it cannot be fairly rejected without careful examination of the natural facts upon which it is stated to rest. Observation alone can determine what is true and what is false in phrenology; but we much wish, in reference to this point, that inquirers would seek to determine, as a preliminary step, the just *method of investigation,* and the *value of evidence* in relation to cerebral physiology; for as observed, apparently with justice, by Dr. Combe, "it has been the want of a sound method of investigation, and not any inherent difficulty in the subject, or any marvellous complexity of function, which has hitherto constituted the chief obstacle to success. Nature's laws and operations rarely remain wholly inaccessible to well-directed and persevering inquiry, and they seem to be a maze of confusion and contradiction only when considered in a wrong point of view, or when examined apart from their natural relations to each other."

There are many reasons, indeed, why Gall's physiology of the brain should no longer experience neglect from the members of our profession; amongst others may be adduced the circumstance that, if true, it must of necessity form the basis of all rational treatment of the insane; and already many experienced and successful practitioners in this department testify to the advantages they derive from this physiology. The whole question is fundamentally one of fact, and as such it should be regarded; all mere *opinion* is obviously inadequate to the formation of solid conclusions. Dr.

Combe says, "Where mere opinion is brought against what I know, from direct, careful, and repeated observation, to be *clear* and *positive facts*, no matter how eminent the source of the opinion may be, I stand firm and unmoved, because Nature is at my back, and I have the fullest assurance that she commits no mistakes, and is never inconsistent; and I know that, on appeal being made, she will be found to speak the same language to-day as yesterday, or as a thousand years ago, and to bear out all I have advanced, if I have really been accurate in my observations." This language expresses sound philosophy, it must be admitted, whatever be thought of the particular subject to which it is applied.

Dr. Combe, in maintaining the necessity of inquiry, observes, "You cannot with safety continue to neglect this inquiry; because the truth is advancing while you are inactive, and you are not in possession of any other knowledge which can warrant you in condemning the claims of phrenology untried. In common fairness you are bound, at least, to make yourselves acquainted with both sides of the question, and to suspend your judgment until you have done so." We readily adopt this sentiment.

The address itself needs no other commendation at our hands than that, in thought, style, and illustration, it is characteristic of Dr. Combe.

